# Serious electronic games as behavioural change interventions in healthcare-associated infections and infection prevention and control: a scoping review of the literature and future directions

**DOI:** 10.1186/s13756-016-0137-0

**Published:** 2016-10-12

**Authors:** Enrique Castro-Sánchez, Yiannis Kyratsis, Michiyo Iwami, Timothy M. Rawson, Alison H. Holmes

**Affiliations:** 1 NIHR Health Protection Research Unit in Healthcare Associated Infection & Antimicrobial Resistance at Imperial College London, Hammersmith Campus, du Cane Road, London, W12 0NN UK; 2 Health Services Research & Management Division, School of Health Sciences, City University London, London, UK

**Keywords:** Serious game, Gamification, Healthcare-associated infection, Implementation, Adoption, Scoping study

## Abstract

**Background:**

The uptake of improvement initiatives in infection prevention and control (IPC) has often proven challenging. Innovative interventions such as ‘serious games’ have been proposed in other areas to educate and help clinicians adopt optimal behaviours. There is limited evidence about the application and evaluation of serious games in IPC. The purposes of the study were: a) to synthesise research evidence on the use of serious games in IPC to support healthcare workers’ behaviour change and best practice learning; and b) to identify gaps across the formulation and evaluation of serious games in IPC.

**Methods:**

A scoping study was conducted using the methodological framework developed by Arksey and O’Malley. We interrogated electronic databases (Ovid MEDLINE, Embase Classic + Embase, PsycINFO, Scopus, Cochrane, Google Scholar) in December 2015. Evidence from these studies was assessed against an analytic framework of intervention formulation and evaluation.

**Results:**

Nine hundred sixty five unique papers were initially identified, 23 included for full-text review, and four finally selected. Studies focused on intervention inception and development rather than implementation. Expert involvement in game design was reported in 2/4 studies. Potential game users were not included in needs assessment and game development. Outcome variables such as fidelity or sustainability were scarcely reported.

**Conclusions:**

The growing interest in serious games for health has not been coupled with adequate evaluation of processes, outcomes and contexts involved. Explanations about the mechanisms by which game components may facilitate behaviour change are lacking, further hindering adoption.

**Electronic supplementary material:**

The online version of this article (doi:10.1186/s13756-016-0137-0) contains supplementary material, which is available to authorized users.

## Background

Healthcare-associated infections (HCAIs) affect millions of patients worldwide with significant economic and human costs [1, 2]. To address this challenge, healthcare organisations have implemented multiple improvement strategies with varying success [3]. These include educational programmes [4, 5], performance feedback [6] and guidelines [7], often ‘bundled’ [8].

However, engaging healthcare workers (HCWs) in sustained best practice remains a challenge. For such reason, the exploration of individual [9] and social motivations [10, 11], and the consideration of organisational contexts [12] has been suggested as beneficial for the effective adoption of behaviour change strategies.

The use of innovative interventions such as serious games to encourage optimal clinical behaviours has received increasing attention for their potential to overcome engagement deficits [13]. A ‘serious game’ is defined as an ‘interactive computer application, with or without significant hardware component, that has a challenging goal, is fun to play and engaging, incorporates some scoring mechanism, and supplies the user with skills, knowledge or attitudes useful in reality’ [13]. Serious games have already been used in clinical medicine, surgery and public health with successful results [14–16]. Seemingly, game users enjoy interacting with games because they can fulfil psychological needs such as control, autonomy and a sense of achievement [14].

Despite increasing evidence for the role of serious games in several fields, there remains a paucity of data supporting their application within infection prevention and control (IPC). To explore whether serious games could promote HCW behaviour change in this crucial area, we conducted a scoping study aiming to synthesise relevant evidence and identify knowledge gaps in the development and evaluation of game-based interventions.

## Methods

### Scope

We largely followed the framework suggested by Arksey and O’Malley [17] combining literature searches, iterative study selection and data extraction, qualitative analysis and identification of implications for future research.

### Application of analytic framework

**Fig. 1:**
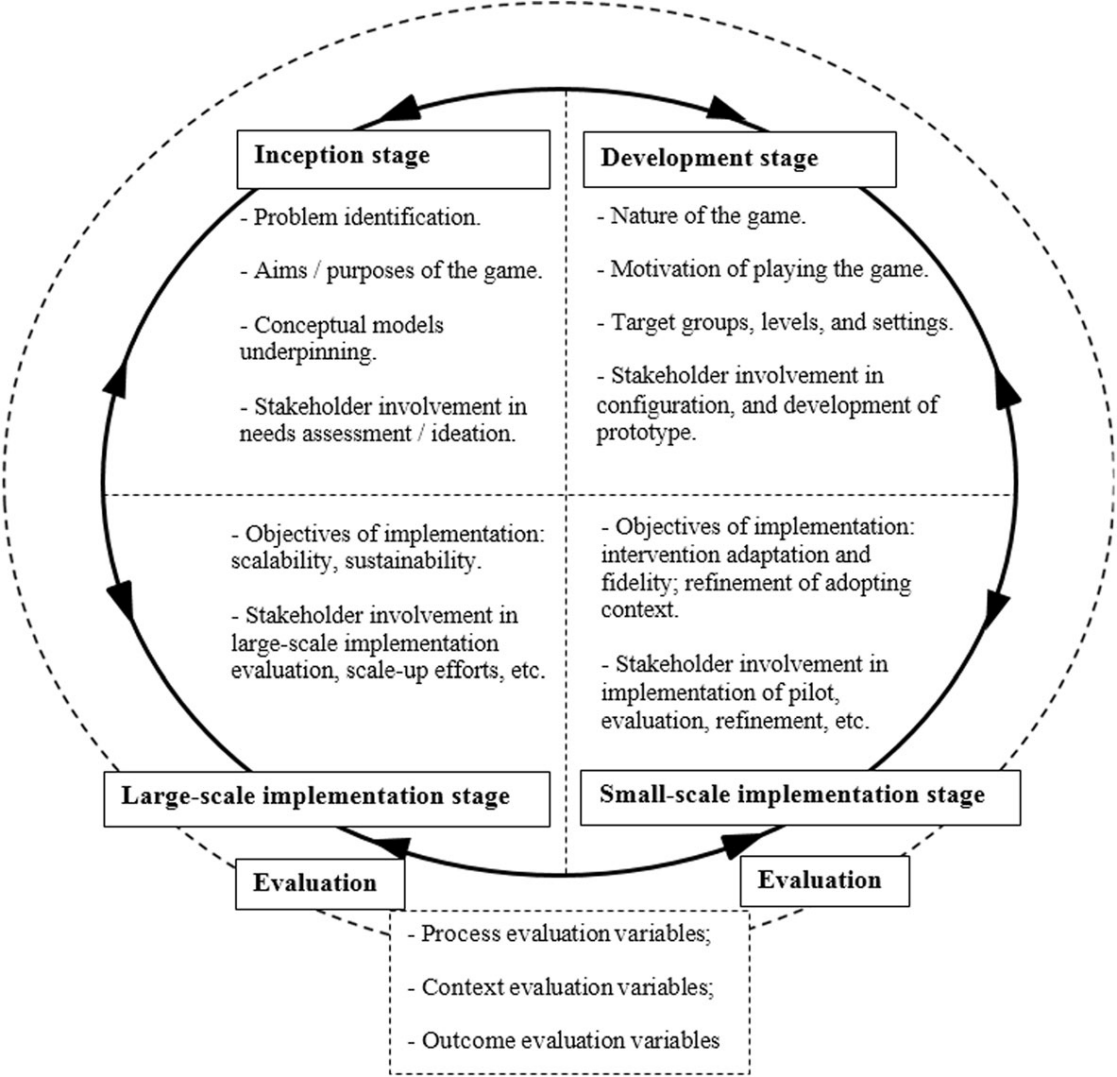
Analytic framework for mapping the games along the stages of intervention formulation and evaluation

The complexity and heterogeneity of serious games may explain the scarcity of evaluative approaches [18]. Although a framework for the evaluation of serious games has been proposed [19], it was constructed following expert opinions and has yet to be validated; in addition, it lacks consideration to the implementation stage for interventions, which have been demonstrated as important for consideration in other models for the development of behaviour interventions [20]. For this reason, we chose to adopt an analytic framework based on the Stage Model for Behavioural Intervention Development [20]. The framework comprises four stages: i) inception, ii) development, iii) small-, and, iv) large-scale implementation, which are iterative and bidirectional (i.e. informing each other) rather than linear and sequential (see Fig. 1).

Although evaluation occurs mainly at the later cycle stages, it is also closely linked to, and draws upon, knowledge and information obtained in preceding stages (see Table 1 for more detail).

**Table 1:** Evaluation Variables

• *Process evaluation variables*: a) evaluation methods: sampling, duration, data collection (pre-/in-/post-game, use of frameworks, comparison, nature of evaluators (multiplicity, independency), reflexivity on risk of bias (roles of researchers, internal/external validity), etc.; b) identification of active component of game intervention (explanation about mechanisms by which game components may facilitate intended changes); game concept; learning model [18]; behaviour change model [30]; engagement with the game, etc.
• *Context evaluation variables*: a) inner context: characteristics of adopting individual/organisation/setting; structure (e.g. size of organisation); culture (e.g. organisational commitment); cognitive (e.g. psychological safety); characteristics of participants (gamers) such as socio-demographic (e.g. age, gender, ethnicity); expertise/professional background, position, (previous gaming) experience; personality; learning styles; game skills/attitudes; intrinsic/extrinsic motivation, etc.; b) outer context (macro-context): incentives and mandates, networks, environmental stability, socio-political climate, etc [18].
• *Outcome evaluation variables*: a) actual use: users’ perceptions about usability, acceptance, and/or attractiveness of the game intervention; users’ knowledge, attitude, behavioural change; clinical effectiveness, efficacy (e.g. dose – effect relations), cost-benefit, unintended outcomes, etc.; b) application: scalability, sustainability, fidelity [31], adaptation, etc.

### Data sources and searches

We interrogated the electronic databases Ovid MEDLINE, Embase Classic + Embase, PsycINFO, Scopus, and the Cochrane Library Database. Additionally, we used Google Scholar to identify additional references. Search terms were developed and refined following a pilot literature scan and through team discussion in November 2015. The following search terms were used and truncated where necessary, with appropriate MeSH terms where possible: ‘serious game’, ‘gamification’, ‘video game’, ‘computer game’, ‘simulation game’, ‘virtual reality’, ‘infection’, ‘hand hygiene’. Inclusion criteria included: 1) papers published up to present, 2) English language, 3) focus on inception, development, implementation, and/or evaluation of serious game interventions in IPC or HCAI prevention and 4) interventions aimed at HCWs (doctors, pharmacists or nurses). Additionally, the exclusion criteria were: 1) interventions aimed at patients or citizens, and 2) non-intervention studies. See Table 2 for search string, with further details in Additional file 1. We ran the search in December 2015.

**Table 2:** Search string (Ovid MEDLINE, Embase Classic + Embase), 10 Dec 2015^a^

(((serious gam* or gamification or (video gam* or video game [MeSH]) or computer gam* or simulation gam* or (virtual reality [MeSH] or virtual realit*)) and (infection [MeSH] or infection*)) not HIV).af.	

*af* All Fields

^a^On 11 December 2015, we supplemented the search string with ‘hand hygiene’

### Study screening and selection

The Covidence [21] platform was used for study screening and selection. Two researchers (ECS/MI) concurrently and independently selected studies (i.e. title and abstract screening, full text review). A third researcher (TMR) resolved disagreements, with final consensus achieved through discussion.

### Data extraction, synthesis, and analysis

A data extraction form was populated with relevant information from each source. As this was a scoping study, we chose not to perform quality assessment of the papers. Inter-rater reliability of analysis was ensured by the independent involvement of the main researchers in assessment.

## Results

### Studies included and data extraction

Our initial search identified 1125 papers. We removed 160 duplicates and excluded 942 papers in the title and abstract screening stage. 23 papers were assessed for full-text eligibility. Subsequently, 19 references were excluded due to irrelevancy (e.g. not focused on games *per se*, not digital games, not infection focus, not aiming at HCWs, not primary research), and full text unavailability. Our review finally analysed four sources (Additional file 2). The authors of conference abstracts and proceedings were contacted to provide information regarding their experiences, together with their presentations. We received two responses with presentation slides.

Results from the descriptive and analytical exercises are shown in Table 3 and Additional file 3 respectively.

**Table 3:** Descriptive overview of studies selected

Authors, Year, Ref	Study 1 Sax and Longtin 2011 [22]	Study 2 Vázquez- Vázquez et al. 2011 [23]	Study 3 Castro-Sánchez et al. 2014 [24]	Study 4 Venier et al*.* 2015 [25]
Type of paper	Conference (presentation)	Conference	Journal article	Conference (presentation)
Origin of the paper	Switzerland (/Canada)	Spain	England	France
Lead (type of organisation)	University hospital	Regional Patient Safety Observatory (Spain)	University	Coordination centre (fighting nosocomial infections)
Paper focus^a^	i), ii) and iii) – Inception, scoping; design, development; pretesting, refinement; and successful launching described. No evaluation of implementation done besides pretesting.	i) and ii) - Inception, scoping; design, development, implementation (launching), but no pretesting/pilot, evaluation done.	i) and ii) – Inception, scoping; design, development. No pretesting. Future evaluation provided.	i), ii) and iv) – brief description about inception, scoping, development, and more focusing on description about implementation of a large scale survey, and its evaluation. No pretesting/pilot studies.
Name of game	Story-based serious game	Serious for hand hygiene training.	‘On call: antibiotics’	Flu.0
Description of game intervention	Game users can decide where to use hand hygiene and disposable gloves using story-based serious game in which 2 doctors are interacting with different patients during ward rounds. Emotional engagement, role identity development through medical specific distracting plot, and mental simulation. Immediate feedback messages and tracking mechanism of results are also incorporated.	Promotion of hand hygiene using WHO’s ‘Five Moments for Hand Hygiene’ with a ludic approach. A non-risk environment was created without any adverse effects from actions of game users, who have to decide when and how hand hygiene should be performed in a 3D setting with different hotspots. Every decision is followed by feedback to strengthen success or to explain why game users performed incorrectly. Low cognitive erosion to keep the playability.	Serious game for antimicrobial prescribing decisions in virtual hospital patients. Prescribers receive clinical information and have to make diagnostic and therapeutic decisions. They get immediate feedback on performance and wider impacts of prescribing decisions. Personalisation/scores/leader boards and difficulty enhancement mechanisms incorporated in the game to sustain engagement.	Serious game for nurses and doctors to educate 8 key points to know and to do when dealing with one or more patients with flu.

^a^i) inception, scoping, ideation; ii) design, development, configuration; iii) small-scale implementation (pretesting/piloting), refinement; iv) large-scale, wide implementation, sustainability

### Thematic categories

The following themes were identified: a) a nascent field; b) suboptimal user engagement; and c) limited process, context, and outcome evaluations to inform applicability of interventions.

- *An initial look at a nascent field*

All studies were conducted in European countries. No study was carried out before 2011. Our search strategy yielded few published papers, but a high proportion of conference abstracts (3/4).

Most games focused on inception or development and implementation, with pending evaluation [22–24]. Only Flu.0 [25] reported on evaluation.

One source discussed timers as engagement mechanism [24]. Other intrinsic motivators such as scores and leader boards [24] or benchmarking [22, 23] were rarely explicit. A ludic approach to WHO’s Five Moments for Hand Hygiene was employed in one study [23]. No intervention incorporated reward mechanisms. Three of the four studies [22–24] aimed at learning and behavioural outcomes whereas Flu.0 [25] focused on learning and attitudinal outcomes. One intervention encompassed primary and secondary care [23], with the rest focusing solely on hospitals. Hand hygiene was the most frequently selected topic [22, 23].

b) *Suboptimal and user engagement in conception and development stages*

Technical experts were involved in two studies (2/4), concerning hand hygiene [23] and antimicrobial stewardship [24]. However, no study explicitly involved external marketers. Equally, no potential game users were included in preliminary needs assessments. Frontline health professionals co-operated in a usability test with ‘think-aloud’ protocols [22], as well as designing virtual patients in another intervention [24].

We found limited user-centred evaluation or summative evaluation [26] (e.g. comparison between control and intervention groups, or triangulated with other data sources).

c) *Limited process, context, and outcome evaluations to inform the applicability of game interventions*

The type of professionals included varied, from doctors [22] to a mixture of nurses and doctors [25] as well as others [23, 24]. Only one study targeting multi-professional groups stratified outcomes by participants’ profession [25]. Effectiveness was reported in two studies, and included changes in users’ perception, knowledge, attitude, and behaviour driven by the intervention. However, no study assessed clinical effectiveness. Intervention fidelity was only dealt with in one study. Implementation variables were either rarely mentioned (in the case of fidelity and unintended outcomes) or absent (as in sustainability). Context evaluation variables such as size of the organisation or participant’s characteristics, as well as process evaluation variables including communication received scant attention.

Regarding other variables such as scalability, Sax and Longtin [22] inferred the applicability of the game to hand hygiene observers, a different target group. Vázquez-Vázquez et al. [23] discussed the potential expansion of the intervention to patients. The Andalusian game developers proposed to export the intervention to other Spanish-speaking countries [23]. However, neither of these was empirically confirmed nor were the interventions culturally adapted.

None of the studies included economic analyses, and just one paper reflected upon the topic at all [24]. Such evaluation would nonetheless be required to assess the viability of mass game deployment.

## Discussion

### Principal findings

In summary, we identified four published experiences related to game-based behavioural interventions in IPC. The absence of studies before 2011 together with the high number of conference papers (3/4) contributing to our review reinforces the notion of a budding research field.

With the information available, game-based behavioural interventions in IPC did not seem adequately evaluated, and generally lacked appropriate control groups. The experience of the game for the participating individuals appeared to be equally neglected in evaluations. These deficits would particularly affect absolute intervention effects, internal validity and generalisation of findings, and the subtle relationships between the games and implementation settings.

Outcome variables including fidelity, unintended outcomes, or sustainability were scarcely reported. Scalability of game-based interventions needs to be explored further. For example, analysis of outcomes on those beyond the target groups would enhance the internal validity of the intervention. Moreover, no study was found that conducted economic analyses of their intervention. The uncertainty about economic aspects [27] would undoubtedly affect estimates about the sustainability of game-based interventions. The absence of sustainability assessments appears to be particularly concerning, in view of the suggested appeal of serious electronic games as behaviour change interventions, and the rapid yet constant replacement of technologies and software.

### Limitations

Our study has various methodological and practical limitations. Relevant studies were small and most papers were presented at conferences, limiting the access to detailed information about the experiences reported. We tried to reduce the impact of such limitation by obtaining information directly from study authors. Our scoping study did not include non-English or grey literature. We focused on scientific publications, inevitably excluding commercial or non-academic papers that may report about games developed within our target area. However, it is unlikely that clinically-focused interventions would be unreported in healthcare conferences or journals. The analytic framework that we used may also have weaknesses, potentially neglecting the informal knowledge transfer that might also play a role in the conception and evaluation of serious games as intervention. However, we opted to use such framework in view of its comparative advantage over the untested tool proposed by Graafland et al [19] and our interest in exploring the implementation features of the experiences identified.

### Future directions

Our scoping study indicates that experiences and research into digital games as behaviour change tools in HCAIs and IPC is an emergent field. The experiences reported focused on a narrow set of clinical areas and scenarios, although the limited number of studies included in our study prevents any firm conclusions. The possibilities afforded by games to explore alternative scenarios and immediate consequences of decisions could be exploited much more efficiently by healthcare organisations. The confluence of technologies such as affordable virtual reality consumer goggles [28] or haptic devices [29] with interventions such as serious games can herald a new era for healthcare training.

For example, role-playing scenarios related to antimicrobial decisions could serve to make explicit the influences exerted on antimicrobial prescribing from other clinicians and patients. Additionally, serious games centred on strategic decision-making may increase practitioner preparedness related to containment of outbreaks of highly virulent pathogens. The same platform may offer lessons about risk communication and management. Games could also enhance the training on traditional yet mechanical IPC tasks such as donning of protective suits or decontamination of surfaces. Personnel involved in the response to future international health emergencies could benefit from culturally-sensitive and accurate serious games that would facilitate their rapid immersion in the assignment.

## Conclusions

Whilst there is growing interest in using serious games in health as valuable adjunct to conventional education, training and behaviour change interventions Perhaps due to the immaturity of the field effectiveness, process, context, and outcome evaluation are still missing and methodological aspects can generally be much improved. Future experiences should incorporate multi-level perspectives including data from the game per se as well as individual, team, organisation, and system levels, to capture more subtle interactions between the interventions and implementation contexts. This rich mesh of feedback channels may clarify the mechanisms by which game components may facilitate behaviour change. With antimicrobial resistance attracting attention in recent years from international campaigns and national policies and efforts to include antimicrobial stewardship at high-level decision fora, we suspect that this area is likely to flourish globally.

## Additional files

Additional file 1: Search strings in five electronic databases and Google Scholar. Search strings in Ovid MEDILINE, Embase Classic + Embase, PsycINFO, Scopus, The Cochrane Library Database, and Google Scholar. (DOCX 43 kb)
Additional file 2: Flow chart depicting the selection of studies. Flow chart depicting the selection of studies in the scoping review. (DOCX 86 kb)
Additional file 3: Results of the application of the proposed analytic framework. Results of the four studies using the proposed analytic framework of intervention formulation and evaluation. (DOCX 61 kb)

## Abbreviations

HCAI: Healthcare associated infection
HCWs: Healthcare workers
IPC: Infection prevention and control
