# Supplementary material for: Serious electronic games as behavioural change interventions in healthcare-associated infections and infection prevention and control: a scoping review of the literature and future directions
Source: Antimicrob Resist Infect Control. 2016 Oct 12;5:34. doi: 10.1186/s13756-016-0137-0 (PMC5062920; doi:10.1186/s13756-016-0137-0)
Supplement: Additional file 1: — Search strings in five electronic databases and Google Scholar. Search strings in Ovid MEDILINE, Embase Classic + Embase, PsycINFO, Scopus, The Cochrane Library Database, and Google Scholar. (DOCX 43 kb) [file 13756_2016_137_MOESM1_ESM.docx]

## **Additional files**

**File name: Additional file 1**

Title: **Additional file 1**. Search strings in five electronic databases and Google Scholar.

Description of data: Search strings in Ovid MEDILINE, Embase Classic+Embase, PsycINFO, Scopus, The Cochrane Library Database, and Google Scholar.

*Search string in Ovid MEDILINE, Embase Classic+Embase, PsycINFO:*

Searched on 10 December 2015

1 serious gam*.mp.

2 gamification.mp.

3 video gam*.mp. or exp video game/

4 computer gam*.mp.

5 simulation gam*.mp.

6 exp virtual reality/ or virtual realit*.mp.

7 1 or 2 or 3 or 4 or 5 or 6

8 exp infection/ or infection*.mp.

9 7 and 8

10 (((serious gam* or gamification or (video gam* or video game) or computer gam* or simulation gam* or (virtual reality or virtual realit*)) and (infection or infection*)) not HIV).af.

11 limit 10 to english language

Ovid MEDLINE(R) <1946 to November Week 3 2015>

Embase Classic+Embasse <1947 to 2015 December 09>

PsycINFO <1806 to December Week 1 2015>

Searched on 11 December 2015

12 hand hygiene.mp.

13 7 and 12

14 limit 13 to english language

Ovid MEDLINE(R) <1946 to November Week 3 2015>

Embase Classic+Embasse <1947 to 2015 December 10>

PsycINFO <1806 to December Week 2 2015>

*Search string in Scopus:*

Searched on 10 December 2015

( ( ( KEY ( infection ) )  AND  (  ( serious  gam* )  OR  ( gamification )  OR  ( video  gam* )  OR  ( computer  gam* )  OR  ( simulation  gam* )  OR  ( virtual  reality ) ) )  AND NOT  ( hiv ) ) AND  ( LIMIT-TO ( LANGUAGE ,  "English" ) )

Scopus (1960 to present)

Limitation of subject areas: Health Sciences, Social Sciences & Humanities

Searched on 11 December 2015

( ( ( KEY ( hand hygiene ) )  AND  (  ( serious  gam* )  OR  ( gamification )  OR  ( video  gam* )  OR  ( computer  gam* )  OR  ( simulation  gam* )  OR  ( virtual  reality ) ) )  AND NOT  ( hiv ) ) AND  ( LIMIT-TO ( LANGUAGE ,  "English" ) )

Scopus (1960 to present)

Limitation of subject areas: Health Sciences, Social Sciences & Humanities

*Search string in The Cochrane Library Database:*

Searched on 10 December 2015

#1 serious gam*:kw (Word variations have been searched)

#2 gamification:kw (Word variations have been searched)

#3 video gam*:kw (Word variations have been searched)

#4 MeSH descriptor: [Video Games] explode all trees

#5 computer gam*:kw (Word variations have been searched)

#6 simulation gam*:kw (Word variations have been searched)

#7 virtual realit*:kw (Word variations have been searched)

#8 #1 or #2 or #3 or #4 or #5 or #6 or #7

#9 infection*:kw (Word variations have been searched)

#10 MeSH descriptor: [Infection] explode all trees

#11 HIV:kw (Word variations have been searched)

#12 (#8 and (#9 or #10)) and not #11

The Cochrane Library Database (all years)

Searched on 11 December 2015

#13 hand hygiene:kw (Word variations have been searched)

#14 MeSH descriptor: [Hand Hygiene] explode all trees

#15 (#8 and (#13 or #14)) and not #11

The Cochrane Library Database (all years)

*Search string in Google Scholar:*

Searched on 14 December 2015

(serious gam* or gamification or (video gam* or video game/) or computer gam* or simulation gam* or (virtual realit* or virtual reality/)) and (infection* or infection/)) not HIV)
