# Supplementary material for: Serious electronic games as behavioural change interventions in healthcare-associated infections and infection prevention and control: a scoping review of the literature and future directions
Source: Antimicrob Resist Infect Control. 2016 Oct 12;5:34. doi: 10.1186/s13756-016-0137-0 (PMC5062920; doi:10.1186/s13756-016-0137-0)
Supplement: Additional file 2: — Flow chart depicting the selection of studies. Flow chart depicting the selection of studies in the scoping review. (DOCX 86 kb) [file 13756_2016_137_MOESM2_ESM.docx]

**File name: Additional file 2**

Title: **Additional file 2**. Flow chart depicting the selection of studies.

Description of data: Flow chart depicting the selection of studies in the scoping review.

1115 studies identified through 5 electronic databases: Ovid MEDLINE, Embase Classic+Embase, PsycINFO, Scopus, and Cochrane Library

160 duplicates removed via Covidence

942 studies excluded as inclusion criteria not met after review of title / abstracts

965 studies screened

10 studies identified through Google Scholar

Identification

Screening

23 studies assessed for eligibility

19 studies excluded following review of full-text:

7 Not focused on games;

1 No electronic games;

2 Not focused on infection;

6 Not focused on healthcare workers;

1 Not primary research;

2 Full text not available

Eligibility

4 studies selected

Included
