# Supplementary material for: Serious electronic games as behavioural change interventions in healthcare-associated infections and infection prevention and control: a scoping review of the literature and future directions
Source: Antimicrob Resist Infect Control. 2016 Oct 12;5:34. doi: 10.1186/s13756-016-0137-0 (PMC5062920; doi:10.1186/s13756-016-0137-0)
Supplement: Additional file 3: — Results of the application of the proposed analytic framework. Results of the four studies using the proposed analytic framework of intervention formulation and evaluation. (DOCX 61 kb) [file 13756_2016_137_MOESM3_ESM.docx]

**File name: Additional file 3**

Title: **Additional file 3**. Results of the application of the proposed analytic framework.

Description of data: Results of the four studies using the proposed analytic framework of intervention formulation and evaluation.

| **Stage** | Element of analysis | Study 1  Sax and Longtin [22] | Study 2  Vázquez- Vázquez et al. [23] | Study 3  Castro-Sánchez et al. [24] | Study 4  Venier et al. [25] |
| --- | --- | --- | --- | --- | --- |
| **i) Inception, scoping, ideation** | Problem identification | Doctors tend to underperform hand hygiene practice. | World Health Organization guidelines (2009) emphasises training on hand hygiene (Five Moments for Hand Hygiene) to all health professionals, and encourages organisations to develop & identify innovative training methods. | Antimicrobial stewardship interventions to improve the quality of prescribing with mixed results, therefore, attention to behavioural and social influences deemed vital for sustainability. | Knowledge and practices on influenza diagnosis, treatment, and infection control need improvement. |
|  | Aims^a^ of the game | Knowledge attainment;  Knowledge sustainment;  Behaviour change;  Skill improvement;  Training | Knowledge attainment;  Attitudinal change;  Behaviour change;  Skill improvement;  Training;  ‘Edutainment’ | Knowledge attainment;  Knowledge sustainment;  Behaviour change;  Skill improvement;  Training;  ‘Edutainment’ | Knowledge attainment;  Attitudinal change;  Skill improvement |
|  | Purpose of the game | Development of a natural immersive environment for improving hand hygiene training and compliance amongst ward doctors. | Training of healthcare workers and citizens on hand hygiene. | Supporting antimicrobial stewardship for prescribers and sustain improvement through behavioural and social influences on prescribing. | To educate 8 key points to know and to do when dealing with one or more patients with flu. |
|  | Conceptual models underpinning^b^ | Not explicitly reported. | Not explicitly reported. | Not explicitly reported. | Not explicitly reported. |
|  | Stakeholder involvement in needs assessment/  ideation^c^ | Not reported. | Not reported about the stakeholder involvement in needs assessment, but in ideation:  1) Constant supervision by preventive medicine doctors, hand hygiene experts to ensure game information is correct &clear; 2) by psychologists and educators to ensure appropriateness of methodology for knowledge transfer. | Not reported. | Not reported. |
| **ii) Design, development (prototype building), configuration** | Nature of the game (type of the game; platform types; topic) | Immersive environment for training and behaviour change | Simulation game | Serious game | Serious game |
|  |  | Computer | Online | Smartphone, tablet, computer | Online or downloaded |
|  |  | Hand hygiene | Hand hygiene | Prudent use of antimicrobials | Flu |
|  | Motivation of playing the game^d^ | Intrinsic (benchmarking and results tracking embedded), but implicit | Intrinsic (benchmarking knowledge progress over time embedded), but implicit | Intrinsic (competitive elements embedded), but implicit | Extrinsic, but implicit |
|  | Target groups, levels^e^, and settings | Doctors working on hospital ward. | Healthcare workers in hospitals, primary health care centres (or nursing homes), patient’s homes, and citizens in Andalusia. | Prescribers in hospitals. | Nurses and doctors. |
|  |  | Individual/social. | Individual/social. | Individual/social. | Individual. |
|  | Stakeholder involvement in configuration, and development of prototype^f^ | No collaboration reported. | Involvement of technical experts on usability and simulations to maximise accessibility, attractiveness and playability.  Collaboration with commercial companies not mentioned. | Collaboration with commercial game company reported to develop prototype. Involvement of clinicians (doctors, pharmacists and nurses) in the preparation of virtual patients. | No collaboration reported. |
| **iii) Small-scale implementation (pretesting/piloting), refinement** | Objectives of implementation: intervention adaptation and fidelity; refinement of adopting context. | Adaptation; refinement. | N/A  (Additional mini-games to explore unclear areas mentioned but no further information provided). | N/A  (Adaptation: particular clinical areas, settings, and emerging infections through relatively easy incorporation of new cases into the game) | N/A |
|  | Stakeholder involvement in implementation of pilot, evaluation, refinement, etc. | Pretesting (target group testing, and individual think-aloud protocols such as usability testing) to feed its results into design refinement | N/A | N/A | N/A |
| **iv) Large-scale, system-wide implementation, sustainability** | Objectives of implementation: scalability, sustainability. | N/A  (Sustainability) | N/A  (Scalability: spreading the correct hand hygiene practices amongst healthcare workers and citizens across different levels of care in Andalusia, including all Andalusian health centres) | N/A  (Scalability: potential incorporation of other gamification components such as individual or team-based competition, and social networking; potential use in low and middle-income countries by ensuring light system requirements) | Not reported. |
|  | Stakeholder involvement in large-scale implementation evaluation, scale-up efforts, etc. | N/A | N/A | N/A | Not reported |
| **Evaluation** | Reported stage of evaluation | c) Pretesting but without detailed evidence. | N/A  (Focus on the initiation and development of the serious game, but no evaluation reported). | N/A  (Focus on the development of the game and its use for the game users. No evaluation has been done). | d) Implementation of a large-scale survey |
|  | Process evaluation variables^g^ and their results | Sampling, duration, data collection, use of frameworks, comparison:  No details reported other than doctors working on hospital ward as target group. | Sampling, duration, data collection, use of frameworks, comparison:  N/A | Sampling, duration, data collection, use of frameworks, comparison:  N/A  (Suggests future evaluation methods using a mixed-methods approach: 1) semi-structured interviews (users’ perceptions on experience of the game); 2) two RCTs on efficacy of the game with trainee doctors and medical students, and use of vignettes to assess participants’ knowledge and decision-making pre- and during the intervention; 3) in-game metrics to assess engagement, dose-effect relations, and effects of prompts and nudges on prescriber’s decisions) | Sampling:  264 doctors (213 fellows); 62 senior nurses; 577 student nurses. |
|  |  |  |  |  | Data collection and analysis method:  Change in game users’ opinions about topic (flu), knowledge attained, and intended behaviour /attitude survey (pre- and post-intervention), followed by descriptive analysis. |
|  |  |  |  |  | Comparison:  Detailed analysis of results by professional groups and seniority. |
|  |  | Nature of evaluators (multiplicity, independency): Not reported | Nature of evaluators (multiplicity, independency): N/A | Nature of evaluators (multiplicity, independency): N/A | Nature of evaluators (multiplicity, independency): Not reported |
|  |  | Reflexivity on risk of bias (roles of researchers, internal/external validity):  Not reported by the authors, and not possible to extrapolate (?) based on the information provided. | Reflexivity on risk of bias (roles of researchers, internal/external validity):  No empirical evidence (evaluation) provided, but extrapolated its application to Spanish speaking Latin American countries. | Reflexivity on risk of bias (roles of researchers, internal/external validity):  Not reported by the authors, and not possible to extrapolate (?) based on the information provided. | Reflexivity on risk of bias (roles of researchers, internal/external validity):  Not reported by the authors, and not possible to extrapolate (?) based on the information provided. |
|  |  | Identification of active component of game intervention^h^:  Not reported. | Identification of active component of game intervention^h^:  N/A | Identification of active component of game intervention^h^:  N/A | Identification of active component of game intervention^h^:  Not reported. |
|  |  | Game concept; learning model; behaviour change model:  Not explicitly reported | Game concept; learning model; behaviour change model:  Not explicitly reported | Game concept; learning model; behaviour change model:  Not explicitly reported | Game concept; learning model; behaviour change model:  Not explicitly reported |
|  | Context evaluation variables^i^ and their results | Intrinsic motivation (benchmarking and results tracking embedded), but implicit. | Intrinsic motivation (benchmarking knowledge progress over time embedded), but implicit. | Intrinsic motivation (competitive elements embedded), but implicit. | Extrinsic motivation, but implicit. |
|  |  | No other context variables reported. | No other context variables reported. | No other context variables reported. | No other context variables reported. |
|  | Outcome evaluation variables^j^, and their results | a) Actual use: | a) Actual use:  N/A | a) Actual use:  *Efficacy*: (?) randomised controlled trials with junior doctors and medical students to assess efficacy of the game and the assessment of participants’ knowledge and decision-making pre- and during the intervention are pending; in-game metrics to evaluate dose-effect relations (relationship between duration of game played and prescribing decisions) are pending. | a) Actual use:  Changes in users’ knowledge, awareness, and intended behaviour / attitude amongst game users (nurses and doctors). |
|  |  |  |  | Evaluation on participants’ perceptions about their experience of the game and their perceived behaviour change is pending. |  |
|  |  |  |  | Evaluation of effects of prompts and nudges on prescriber’s decisions by different characters is pending. |  |
|  |  | *Clinical effectiveness*: (?) game users were successfully immersed into real-life hand hygiene challenges, but evaluation of clinical effectiveness is pending. |  |  | *Clinical effectiveness*: (?) knowledge about rapid test for flu and additional precautions increased the most, and 47% doctors/senior nurses and 80% student nurses intended to perform better additional precautions, but evaluation of clinical effectiveness is not reported. |
|  |  | *Cost-benefit*: not reported. |  | *Cost-benefit*: (?) partially considered. | *Cost-benefit*: not reported. |
|  |  | *Unintended outcomes*: not reported. | (*Unintended outcomes*: (?) expected to be no adverse effect mentioned, but no evidence reported). | *Unintended outcomes*: (?) potential negative impact on the productivity of the participants as a result of an excessive amount of playtime mentioned, but its assessment is pending. | *Unintended outcomes*: not reported. |
|  |  | b) Application:  *Adaptation*: (+) pretesting (target group testing, and individual think-aloud protocols such as usability testing) was done to feed its results into design refinement. | b) Application:  *Adaptation*: not reported. | b) Application:  N/A | b) Application:  *Adaptation*: not reported. |
|  |  | *Fidelity of intervention*: (+) all design configurations met;  *Fidelity of implementation process* (pretesting): (?) positive effects were seen, but unclear about fidelity. | *Fidelity of intervention:* not reported;  *Fidelity of implementation process*: not reported. |  | *Fidelity of intervention*: not reported;  *Fidelity of implementation process*: (?) positive effects were seen, but unclear about fidelity. |
|  |  | *Scalability*: (+) applicable to hand hygiene observers. | *Scalability*: (+) potentially applicable to patients; potentially transferable to Spanish speaking countries such as Latin America, but no evaluation results reported; and a second serious game focusing on Safe Medication Practices is under way. |  | *Scalability*: not reported |
|  |  | *Sustainability*: (?) successful launching mentioned, but post-launch evaluation is pending. | *Sustainability*: (?) briefly mentioned but no evidence provided. |  | *Sustainability*: not reported |

Keys: (?): no clear information; (+): positive results; (-): negative results; N/A: not applicable.

^a^Knowledge attainment; knowledge sustainment; attitude change; behaviour change; skill improvement; training; edutainment.

^b^Game concept; learning model [18]; behaviour change model [29].

^c^For example, researchers, funders; potential users; partnerships (internal, external) in needs assessment, etc.

^d^Extrinsic or intrinsic.

^e^Individual, team/group, social.

^f^For example, researchers, designers, developers, potential users; technical/expert/marketing support, professional supervisory support, etc.

^g^Process evaluation variables: a) evaluation methods: sampling, duration, data collection (pre-/in-/post-game, use of frameworks, comparison, nature of evaluators (multiplicity, independency), reflexivity on risk of bias (roles of researchers, internal/external validity), etc.; b) identification of active component of game intervention (explanation about mechanisms by which game components may facilitate intended changes); game concept; learning model; behaviour change model; engagement with the game, etc.

^h^Explanation about mechanisms by which game components may facilitate intended changes.

^i^Context evaluation variables: a) inner context: characteristics of adopting individual/organisation/setting; structure (e.g. size of organisation); culture (e.g. organisational commitment); cognitive (e.g. psychological safety); characteristics of participants (gamers) such as socio-demographic (e.g. age, gender, ethnicity); expertise/professional background, position, (previous gaming) experience; personality; learning styles; game skills/attitudes; intrinsic/extrinsic motivation, etc.; b) outer context (macro-context): incentives and mandates, networks, environmental stability, socio-political climate, etc. [18]

^j^Outcome evaluation variables: a) actual use: users’ perceptions about usability, acceptance, and/or attractiveness of the game intervention; users’ knowledge, attitude, behavioural change; clinical effectiveness, efficacy (e.g. dose – effect relations), cost-benefit, unintended outcomes, etc.; b) application: scalability, sustainability, fidelity [30], adaptation, etc.
